# Mechanism of exogenous enzymes participation in the regulation of digestion and metabolism in model chickens

**DOI:** 10.3389/fvets.2025.1701604

**Published:** 2025-11-25

**Authors:** Vladimir Vertiprakhov, Bayarma S. Dashieva, Alexei M. Grigoriev, Veronika D. Ashikhmina

**Affiliations:** Moscow Timiryazev Agricultural Academy, Moscow, Russia

**Keywords:** trypsin, duodenal chyme, enzyme preparation, fistulated birds, chickens

## Abstract

Experiments were performed on fistulas with duodenal cannula, which can reflect the function of the pancreas and are a model for studying digestion and metabolism when using biologically active additives. The aim of this work is to study the effect of different doses of enzyme preparation prepared from pig pancreas tissue on duodenal enzyme activity, calcium phosphorus, blood biochemical status and productivity in laying hens using fistula technology. The enzyme preparation prepared from pig pancreatic gland tissue was standardized for trypsin activity, which was 187 units/g. The results showed that a dose of 0.01% of the enzyme preparation from the feed weight stimulated metabolism and influenced productive metabolism, increasing egg production by 25.0% compared to the control group, increasing the dose of the preparation led to an increase in trypsin activity in the duodenum, but critically influenced egg formation, reducing its mass by 5.8% and increasing egg laying capacity by 12.5% against the control. Consequently, the use of enzymes should be controlled from the side of mineral metabolism, which can be disturbed at high doses of the drug.

## 1 Introduction

The use of exogenous enzymes to overcome the negative effects of anti-nutritional factors, improve the digestibility of feed components, and increase poultry productivity has become standard practice in poultry feeding. Despite the widespread use of feed enzymes, the response of poultry to their introduction into diets varies, so obtaining consistent results when using enzymes still requires answers to a number of questions. However, these differences in response to enzymes indicate not only existing limitations on their effectiveness, but also the possibility of improving their effectiveness (1). Over the past decades, the chemistry of enzyme target substrates has been studied in greater depth, making it possible to produce specific enzymes for individual substrates. Enzyme biotechnology (fermentation processes, microbiological synthesis) and the molecular biology of enzymatic processes have also developed. Along with biotechnological methods of enzyme production by microbiological synthesis, enzymes prepared from pig pancreatic tissue are used in human medicine. These enzymes have a complex of amylolytic, lipolytic, and proteolytic enzymes and also contain biologically active substances, since the pancreas secretes hormones that affect metabolic functions in the body. Studies conducted to compare different manufacturing technologies for preparations have shown their varying effectiveness (2). Most commercially available products are of animal origin (processed pancreas obtained from slaughterhouses) and contain lipases, alpha-amylase, and proteases. Microbial and plant-derived enzymes appear to be a promising alternative to animal-derived enzymes, but to date, there are no registered drugs containing all enzymes simultaneously for use in clinical practice for the treatment of exocrine pancreatic disorders. The importance of pancreatic enzymes is not limited to digestive functions. For example, trypsin, along with its enzymatic digestive and autocatalytic properties, exhibits exceptional regulatory capacity. In the early 1970s, Rothman recognized trypsin as a hormone (3). Research conducted by Prikhodko et al. (4) showed that proteases stimulate premature maturation of enterocytes. Currently, trypsin is recognized as a potent activator of PAR receptors (5), actively participating in the inflammatory response. Therefore, in order to study the mechanism of action of animal enzymes, it is necessary to use a chicken with a duodenal fistula as a model. Chickens are rarely used as models for studying physiological processes, but since they have a high exocrine function of the pancreas (6), their use as a model for studying digestion is quite justified. The aim of this study is to investigate the effect of various doses of an enzyme preparation made from porcine pancreatic tissue on duodenal enzyme activity, calcium and phosphorus content, blood biochemistry, and the productivity of laying hens using fistula technology.

## 2 Materials and methods

The experimental procedures conducted in this study were approved by the Bioethics Commission of Russian State Agrarian University—Moscow Timiryazev Agricultural Academy (Extract from Minutes No. 46 of 05.05.2025). This approval guarantees compliance with the established standards of ethical treatment of animals and their use in research.

The experiments were carried out on 12 20-week-old chickens of Heissex white cross in accordance with the requirements of the European Convention for the Protection of Vertebrate Animals Used for Experiments or Other Scientific Purposes (ETS No. 123, Strasbourg, 1986). The experiments involved 12 chickens with a pre-established duodenal fistula opposite the point where the three pancreatic ducts and two bile ducts enter the intestine (7). The birds were kept in the vivarium of Russian State Agrarian University—Moscow Timiryazev Agricultural Academy under the condition of feeding and housing in accordance with the requirements for a certain age group and cross of poultry. During the experiment, the chickens were kept in individual cages arranged in three tiers. The duration of daylight hours was set within the following limits: from 20 weeks of age to 25 weeks of age—10 h at an illuminance of 10 lux. Then, every 2 weeks until 40 weeks of age, the duration of daylight hours was increased by 30 min and the illuminance was 25 lux. The chickens were kept indoors at a temperature of 17–19 °C and a humidity of 60–70%.

After recovery of health in chickens after surgery, in 3–5 days we started physiological experiments. The chickens were divided into three groups using the analog method: (1) the control group received a complete feed prepared in accordance with the requirements for this breed of poultry (Table 1); (2) experimental group 1 received the same feed mixed with an enzyme preparation at a dose of 100 mg/kg of feed (0.01% of feed); (3) experimental group 2 received the same feed mixed with an enzyme preparation at a dose of 200 mg/kg of feed (0.02% of feed). The experiment lasted 20 days, during which the number of eggs and feed consumed were recorded daily, every 2–3 days, duodenal chyme was collected for biochemical studies. A complete blood count was performed on the chickens at the end of the experiment. Blood was taken from the wing vein on an empty stomach after 12 h of fasting, but with access to water.

**Table 1:** Nutritive value of mixed fodder for chickens.

Index	Group		
	Control	1 Experienced	2 Experienced
Metabolic energy, kcal/kg	270.0	270.0	270.0
Crude protein, %	17.4	17.4	17.4
Crude fat, %	5.0	5.0	5.0
Crude fiber	5.3	5.3	5.3
Lysine, %	0.70	0.70	0.70
Methionine, %	0.42	0.42	0.42
Calcium, %	3.5	3.5	3.5
Phosphorus, %	0.60	0.60	0.60
M. ash share, %	0.5	0.5	0.5
Enzyme preparation, %	–	0.01	0.02

### 2.1 Physiological experiments

Experiments on poultry were started in the morning on an empty stomach. Chickens were given 70 g of mixed feed and duodenal chyme (0.5–1.0 mL) was collected after 60 and 120 min. It was centrifuged using eppendorf MiniSpin^®^ plus centrifuge (Germany) at 7,000 rpm for 5 min and enzyme activity was determined in the supernatant by diluting it 10 times with 0.9% NaCl solution. The activity of trypsin and alkaline phosphatase and the amount of total calcium and phosphorus in duodenal contents were determined using a semi-automatic biochemical analyzer HTI BioChem SA (HTI Technology, United States).

Trypsin activity was determined by a biochemical method using a with N-benzoyl-DL-arginine-n-n-nitroanilide (BAPNA) as a substrate (8, 9). For this purpose, 450 μL of buffer solution (pH 8.2)—reagent 1—was placed in an Eppendorf tube and 50 μL of reagent 2 containing the trypsin substrate was added. Reagent 2 was prepared as follows: 5.0 mg of N-benzoyl-DL-arginine-n-n-nitroaniline (BAPNA) powder was dissolved in 1.0 mL of dimethyl sulfoxide with constant stirring for 2–3 min. The solution can be stored in a refrigerator at +4 °C for up to 3 months. For analysis, reagents 1 and 2 were mixed in a closed Eppendorf tube and heated for 3 min in a thermostat at +37 °C. After incubation, 10.0 μL of the test material (duodenal chyme, serum) was added, mixed with the reagent mixture, and the reaction was started on a biochemical analyzer. The enzyme determination mode was preset on the device: main filter—405 nm, delay—15 s, measurement time—60 s, control sample—water, coefficient—4,554. Calibration was performed using a TruCal multicalibrator (Di Sys Diagnostic Systems GmbH, Germany). The unit of measurement for trypsin activity was U/L (8, 9), alkaline phosphatase activity and macronutrients - using reagents of the company (DIAVET, Russian Federation).

Biochemical blood analysis (amylase activity, total protein, glucose, triglycerides, cholesterol, alkaline phosphatase, uric acid, calcium, and phosphorus) was performed on an automatic biochemical analyzer BioChem FC-120 (High Technology, Inc., North Attleboro, Massachusetts, United States) using reagent kits from this company.

### 2.2 Statistical methods

The use of these nonparametric methods is justified by their robustness to outliers and violations of the normality assumption, which is critical when analyzing small sample data. Kendall’s *τ* coefficient is the preferred choice for assessing correlations with limited observations, demonstrating higher accuracy in estimating statistical significance (*p*-values) and lower sensitivity to tied ranks compared to alternative nonparametric tests. However, when interpreting the results, potential instability of statistical estimates in small sample conditions must be taken into account. To account for related ranks, Kendall’s coefficient (*τ*-b) is determined using the following formula (10):

$$\tau_{b}=\frac{n_{c}-n_{d}}{\sqrt{\left(n_{0}-n_{1})(n_{0}-n_{2}\right)}}\;$$

where $n_{c}$ – the number of matched pairs, i.e., pairs с *0 < i < j < n*, where $r_{i}<r_{j}$ and $s_{i}<s_{j}$ , or $r_{i}>r_{j}$ и $s_{i}>s_{j}$ ;

$n_{d}$ is the number of unmatched pairs, i.e., pairs with *0 < i < j < n*, where $r_{i}<r_{j}\;$ and $s_{i}>s_{j}$ , or $r_{i}>r_{j\;\;}$ and $s_{i}<s_{j}$ ;

$n_{0}=n(n-1)/2$ – the total number of pairs, where *n* is the number of pairs of observations;

$n_{1}=\sum_{i=1}^{k}\frac{t_{i}(t_{i}-1)}{2}$ ; $n_{2}=\sum_{j=1}^{m}\frac{u_{j}(u_{j}-1)}{2}$ ;

k,m – the number of groups of identical ranks for the variables X and Y;

$t_{i},u_{j}$ – number of ranks in a group of identical ranks, variables X and Y (11).

The Kendall correlation coefficient *τ* will be statistically significant at the level of *α,* if the value of $t_{\text{fact}}=\frac{\tau -0}{s_{\tau }}=\tau \sqrt{\frac{9n(n-1)}{2(2n+5)}}$ exceeds the critical value $\left(t_{1-\alpha }\right)$ in absolute terms. Under the null hypothesis of independence between the examined variables, when *n* > 10, the *τ* statistic follows an approximately normal distribution with expectation equal to zero and standard deviation equal to $s_{\tau }=\sqrt{\frac{2(2n+5)}{9n(n-1)}}$ (11).

In the presence of tied ranks, Spearman’s rank correlation coefficient is calculated using the following formula (11):

$$\rho =1-\frac{\sum_{i=1}^{n}{\left(r_{i}-s_{i}\right)}^{2}}{\frac{1}{6}(n^{3}-n)-(n_{1}+n_{2})}$$

where $r_{i},s_{i}$ – these are the ranks of the i-th object according to the variables X and Y, respectively;

$n_{1}=\frac{1}{12}\sum_{i=1}^{k}(t_{i}^{3}-t_{i});$ $n_{2}=\frac{1}{12}\sum_{j=1}^{m}(u_{j}^{3}-u_{j})$ .

Spearman’s rank correlation coefficient will be significant at the *α* level if $|t_{\text{fact}}|$ is greater than the critical t, where $t_{\text{fact}}=\frac{\rho \sqrt{n-2}}{\sqrt{1-\rho^{2}}}$ , and the $t_{\text{crit}}$ is determined by the significance level α and the number of degrees of freedom df = n-2. If the null hypothesis of the absence of a correlation between features is correct, at *n* > 10 t there is a Student’s t-distribution with the number of degrees of freedom n-2 (11).

## 3 Results

In the experiments, an enzyme preparation made from the tissue of the pancreas of pigs was used.

Trypsin activity in this preparation was determined using the substrate N-benzoyl-DL-arginine-n-n-nitroanilide (BAPNA) (8, 9). The activity was found to be 187 ± 18.6 units/g. The activity of trypsin and alkaline phosphatase, as well as the amount of calcium and phosphorus, were determined in the contents of the duodenum (Table 2).

**Table 2:** Activity of trypsin, alkaline phosphatase, amount of calcium and phosphorus in the duodenal chyme of laying hens of the Hisex white cross (M ± m, *n* = 4).

Index	Group					
	Control		1 Experienced		2 Experienced	
Minutes after feeding	60	120	60	120	60	120
Trypsin activity, u/L	1980±	1926±	2039±	1758±	2,521±	1936±
	65.2	65.9	93.2	143.2	128.9ª	92.2ᵇ
Alkaline	5,236±	5,242±	5,458±	5,058±	5,624 ± 3	4,950±
Phosphatase	344.0	322.8	660.7	424.0	97.5	377.0
Activity, u/L						
Calcium, mmol/L	53 ± 1.8	48 ± 1.6ᵇ	53 ± 2.3	44 ± 2.1ᵇ	46 ± 2.6	43 ± 1.7
Phosphorus, mmol/L	8.4 ± 0.17	8.1 ± 0.21	8.2 ± 0.25	8.2 ± 0.23	7.9 ± 0.17	7.8 ± 0.20

^a^Differences are statistically significant with 1 control group; ^b^differences statistically significant between samples in the same group, at *p* < 0.05.

The results of the studies showed that in the control group there was a decrease in calcium content in duodenal contents 120 min after feeding by 9.4% (*p* < 0.05), which is associated with its metabolism. Trypsin activity increases in duodenal chyme in the first 60 min postprandial period with the addition of enzyme preparation at a dose of 200 mg/kg feed by 27.3% (*p* < 0.05). In this group, a difference between different chyme samples by 23.2% (*p* < 0.05) was noted, which is apparently due to the intestinal intake of exogenous enzymes and pancreatic regulation by the feedback principle (12). A similar trend was observed in the 2nd experimental group when the enzyme preparation was added to the diet of chickens at a dose of 100 mg/kg of feed. Activity of alkaline phosphatase at addition of enzyme preparation in different doses and phosphorus content remain without significant changes according to our data.

A part of energy in the organism in addition to the basic metabolism is spent on physical and mental activity in humans, and in productive animals - on the production of products. In hens the main product is egg, so we analyzed the level of egg production in laying hens and came to the conclusion that the use of exogenous enzymes provides an increase in productivity in animals (Table 3).

**Table 3:** Number of eggs for the experiment and their weight.

Index	Group		
	Control	1 Experienced	2 Experienced
Number of eggs per experiment period, pcs.	16	20	18
Number of eggs per layer, pcs.	4.0	5.0	4.5
Weight of one egg, g	50.9	50.2	49.7

**Figure 1:**
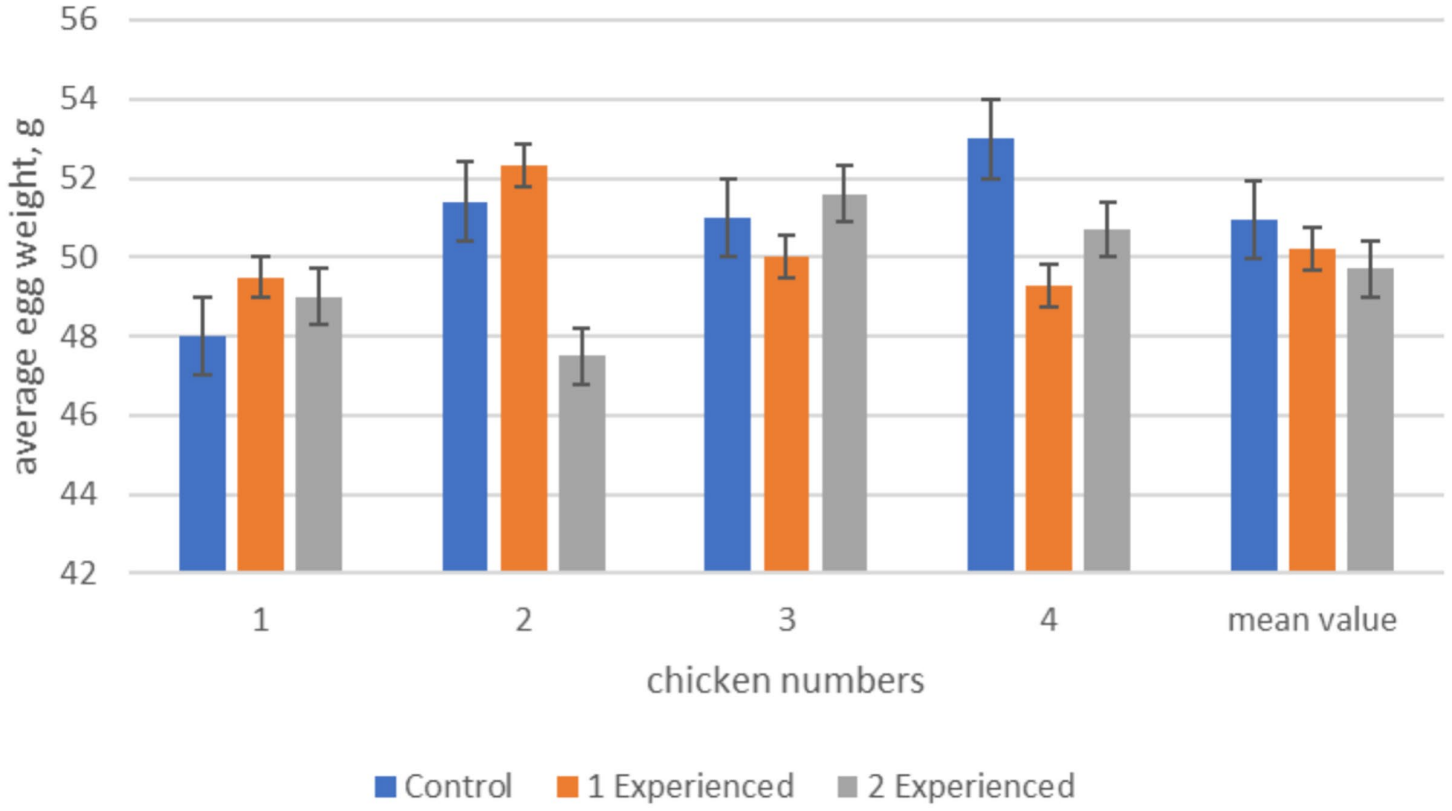
Average egg weight in hens during the experiment period. From this figure it can be seen that statistically significant differences in egg weight are observed between hens No2 and No4 of the experimental 2 and control groups, but the mean value does not have significant differences. Differences between the control and experimental groups 1 were noted only in chicken No4, although on average no statistically significant differences were found.

The results of the studies clearly showed that in the experimental groups the number of eggs laid during the experiment period exceeds the indicators of the control group: in the 1st experimental group (with the addition of the drug at a dose of 100 mg/kg of feed) by 25.0%, in the 2nd experimental group (200 mg/kg of feed)—by 12.5%. Taking into account the higher egg weight in the control group, the indicators of total egg weight prevail in the experimental groups due to the increase in the number of eggs. The drug most effectively increased egg production in the 1st experimental group. This is evidenced by the correlation between trypsin activity and egg production of hens. The results of the egg weight study showed that despite a tendency toward a decrease in egg weight in the experimental group 2 compared to the control group, no statistically significant differences were achieved (Figure 1).

The results of blood biochemical studies allow us to fully assess the animal’s health status and metabolic processes. The data are presented in Table 4.

**Table 4:** Biochemical parameters of chickens’ blood when different doses of enzyme preparation were added to the diet (M ± m, *n* = 4).

Index	Group		
	Control	1 Experienced	2 Experienced
Trypsin activity, u/L	116 ± 10.9	127 ± 6.2	78 ± 9.8 ª
Amylase activity, u/L	604 ± 60.6	425 ± 66.0	897 ± 45.8 ª
Glucose, mmol/L	9.5 ± 0.23	9.8 ± 0.25	11.1 ± 0.10 ª
Total protein, g/L	54.9 ± 2.50	42.5 ± 1.60ª	58.8 ± 3.33
Uric acid, mmol/L	250.6 ± 46.96	114.3 ± 10.50 ª	221.1 ± 25.2
Triglycerides, mmol/L	4.8 ± 0.90	4.9 ± 0.75	1.1 ± 0.5 ª
Cholesterol, mmol/L	5.2 ± 0.60	3.2 ± 0.22ª	5.0 ± 0.35
Alkaline phosphatase, u/L	666 ± 38.5	847 ± 44.1 ª	700 ± 24.5
Calcium, mmol/L	1.7 ± 0.16	1.7 ± 0.04	1.2 ± 0.06 ª
Phosphorus, mmol/L	1.6 ± 0.09	2.6 ± 0.24 ª	1.4 ± 0.10

^a^Differences are statistically significant at *p* < 0.05.

The results showed that the activity of amylase in blood serum increased by 48.5% when the drug was added at a dose of 200 mg/kg of feed, indicating an increase in carbohydrate metabolism, while trypsin, on the contrary, decreased by 32.7% compared to the control group. Trypsin activity responded differently to the doses of the drug added to the diet of chickens. A decrease was observed in 2 Experienced group (78 units/L, *p* < 0.05) and an increase in 1 Experienced group (127 units/L). Total blood protein was increased in the 2 Experienced group (58.8 g/L), decreased in the 1 Experienced group (42.5 g/L, *p* < 0.05). Blood glucose was increased by 16.8% in the 2 Experienced group, which may support metabolism for egg production, but correlates with reduced egg mass - possibly due to stress or overload. In contrast, triglyceride content decreased 77.1%, which may indicate increased consumption for yolk (lipids ~5–6 g/egg), explaining intermediate productivity. Calcium content in the 2nd experimental group decreased by 29.4% compared to the control.

When the preparation was used at a dose of 100 mg/kg of feed, a decrease in blood total protein by 22.6% and uric acid by 54.4% was observed, indicating better protein metabolism without accumulation of waste, which is favorable for stable oviposition. The amount of cholesterol decreased in the 1st experimental group by 38.5%, while phosphorus metabolism was enhanced by increasing alkaline phosphatase activity by 27.2% and blood phosphorus content by 62.5% compared to the control group. Therefore, in this case, metabolism is directed toward catabolism with the formation of energy to enhance egg formation and increase oviposition.

**Figure 2:**
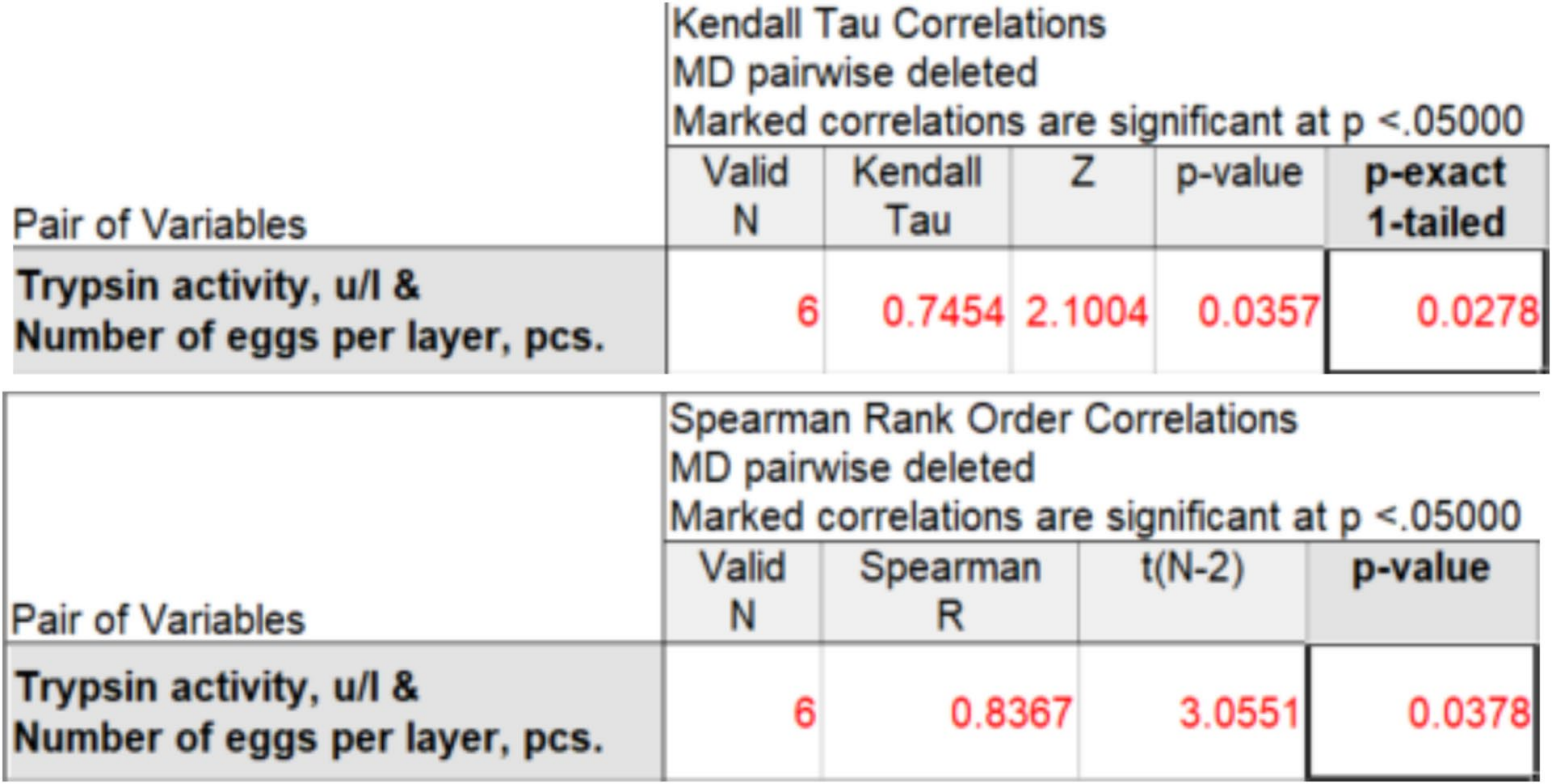
Kendall’s correlation coefficient (*τ*-b) and Spearman’s correlation coefficient (*ρ*) between trypsin activity and egg production at an enzyme preparation dose of 100 mg per 1 kg of feed.

**Figure 3:**
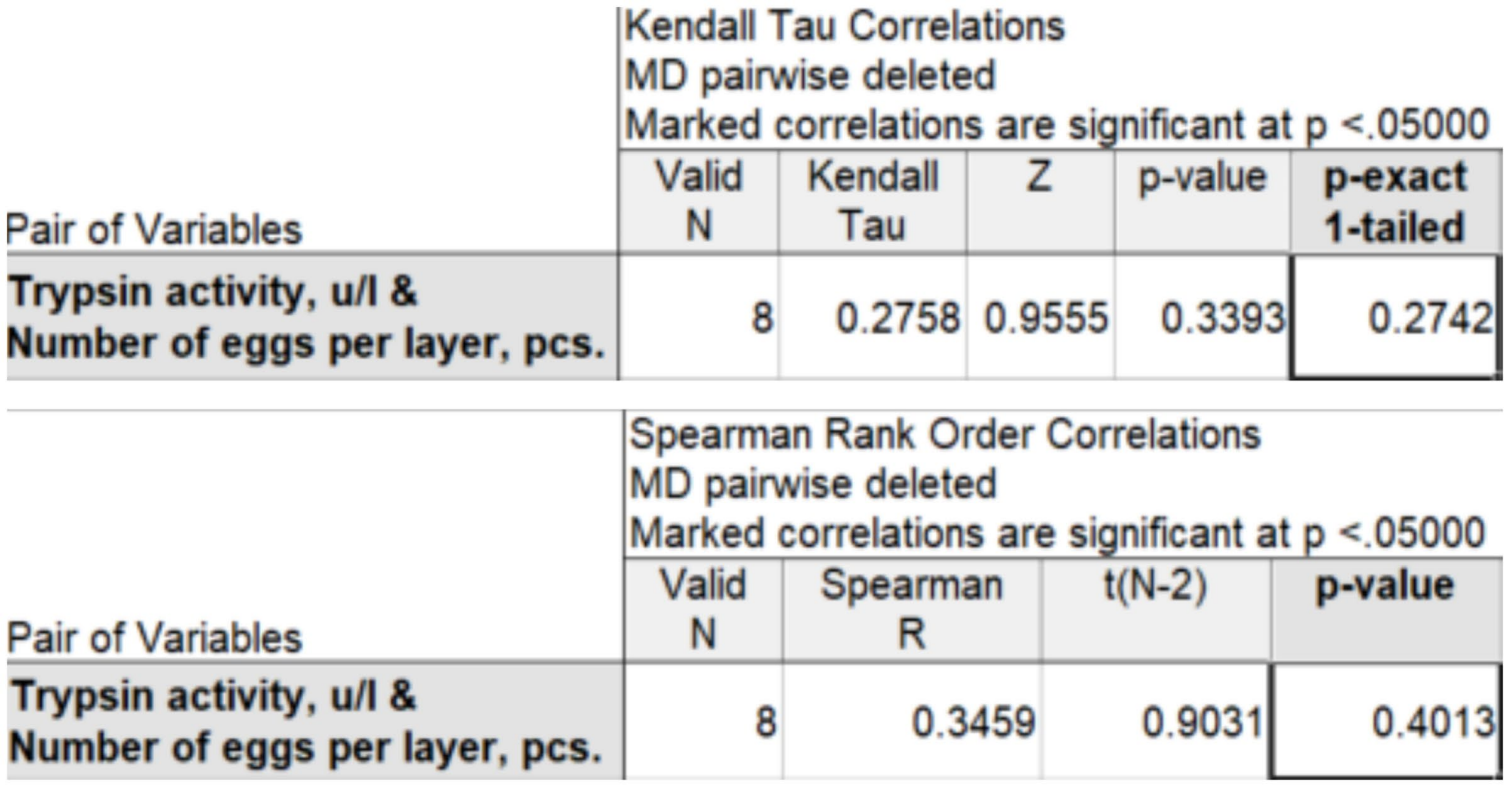
Kendall’s correlation coefficient (τ-b) and Spearman’s correlation coefficient (ρ) between trypsin activity and egg production at an enzyme preparation dose of 200 mg per 1 kg of feed.

Based on data from 6 to 8 observations, Kendall’s rank correlation coefficient (*τ*-b) and Spearman’s correlation coefficient (*ρ*) can be used to determine the strength of the relationship between the studied characteristics. This is statistically acceptable, but conclusions based on such a small number of observations should be presented with great caution. Using the STATISTICA package (version 14.0.0.15), we obtained the values of rank correlation coefficients for two experimental groups: 100 mg (Figure 2) and 200 mg (Figure 3).

When adding 100 mg of the drug per 1 kg of feed, Kendall’s *τ*-b rank correlation coefficient is $\tau_{b}$ =0.745, Spearman’s *ρ* is ρ = 0.837, which indicates a strong positive correlation between trypsin activity and egg production. The rank correlation coefficients $\tau_{b}\;$ and ρ are significant at the 5% level. However, the results should be interpreted with caution, given the limitations due to the small sample size (*n* = 6 days). To confirm the identified relationship and increase the statistical power of the analysis, additional studies over a longer time interval are needed.

To assess the risk of false correlation, an analysis of the autocorrelation structure of each time series was performed. Since the number of observations is small, only the first-order autocorrelation coefficient was determined. The first-order autocorrelation coefficients were low for both traits (trypsin activity: r_1_ = −0.116; egg production: r_1_ = −0.286), indicating a weak sequential dependence between the levels of the series and approximate stationarity. The absence of strong autocorrelation indicates that the identified relationship between trypsin activity and egg production is not an artifact of the general time trend, but reflects a real relationship between the traits. It should be noted that the small sample size does not allow for the application of strict formal tests for stationarity and requires careful interpretation of the results obtained.

At a dose of 200 mg per 1 kg of feed, Kendall’s *τ*-b rank correlation coefficient is $\tau_{b}$ =0.276, and Spearman’s *ρ* is ρ = 0.346, which indicates a weak positive correlation between trypsin activity and egg production. The rank correlation coefficients $\tau_{b}$ = and ρ are statistically insignificant.

## 4 Discussion

Trypsin enhances protein breakdown in the intestine, improving the digestion of amino acids for egg protein synthesis (about 10–12 g protein per egg). In the 1st experimental group, trypsin activity correlates with maximum productivity, possibly due to efficient but not excessive digestion - this reduces total protein in the blood (due to its use in egg laying). In the 2nd experimental group, an early peak of trypsin in the gut leads to increased total blood protein, which improves yolk/albumin mass but reduces egg mass overall (49.7 g)—possibly due to an imbalance with minerals. Overall trend: optimal trypsin (experienced group 1) improves egg counts (+25%), while excessive (experienced group 2) only +12.5%, with a risk of reduced quality. Apparently, calcium absorption in the small intestine fluctuates during the daily cycle of egg formation (13, 14). It is thought to occur mainly in the duodenum and jejunum, and less calcium is absorbed in the ileum (15, 16). Approximately 80% of phosphorus is found in the skeleton as hydroxyapatite. It is released by bone resorption during eggshell calcification, and this excess Pi (17, 18) must be excreted from the body to prevent toxic effects. Maintenance of circulating Pi occurs in the kidney, small intestine, and bone (19) and is primarily regulated by fibroblast growth factor 23 (FGF23); however, PTH and 1,25(OH)2D3 also influence it by affecting calcium homeostasis (20).

Sinclair-Black et al. (21) describe the mechanisms of Ca and P homeostasis in laying hens, emphasizing that daily oviposition requires ~2–3 g Ca per egg, with an increased risk of deficiency in late phases. Our studies have shown that decreased blood Ca (as in the 2nd experimental group) may be associated with increased shell consumption, leading to reduced egg weight. The results showed the role of alkaline phosphatase in mineral mobilization, which is consistent with its increased activity in blood in experimental group 1 for optimum balance. Alkaline phosphatase is involved in dephosphorylation and mobilization of minerals for shell. The increase in blood in experimental group 1 correlates with the highest productivity (20 eggs), indicating improved mineral homeostasis—allowing laying hens to maintain frequent egg laying without exhaustion. In experimental group 2, the stability of phosphatase coincides with intermediate productivity, but possible deficiency. The results of the experiment showed that in the second sample of duodenal chyme the calcium content decreased in the control group from 53 to 48 mmoL/L (*p* < 0.05), in the 2nd experimental group from 46 to 43 mmoL/L, which is lower than the baseline value. Phosphorus level in the 1st experimental group increases to 2.6 mmoL/L (*p* < 0.05), in the 2nd experimental group decreases to 1.4 mmoL/L.

Calcium is the main component of the shell (calcium carbonate ~94%), phosphorus is necessary for balance (optimal Ca: P ratio ~2:1). In the 1st experimental group, stable calcium and increased phosphorus in the blood maintain high productivity and almost normal egg weight (50.2 g)—the preparation improves absorption, minimizing losses in the intestine and providing reserves for daily egg-laying. In the 2nd experimental group, lower blood calcium/phosphorus correlates with lower egg weight (49.7 g) despite higher egg counts (4.5/laying hen)—indicating hypocalcemia, where laying hens use up bone reserves, which can lead to thin shells or reduced productivity over time. Decreases in intestinal absorption (especially in experimental group 2) suggest accelerated calcium absorption, but without compensation, which exacerbates the deficiency at high dose. Huang et al. (22) show that multienzyme linearly increases trypsin activity in the duodenum of laying hens (*p* < 0.05), improving protein digestibility and egg production, which is similar to the results in the second experimental group. However, the greatest effect (optimum) is observed at the average dose, which is consistent with the results of our experiment in experimental group 1 without overload. Yuan et al. (23) in broilers demonstrate a dose-dependent increase in intestinal trypsin (up to +74% at 80 mg/kg protease) but a decrease at 160 mg/kg, which is consistent with the decrease in our experiment at 120 min in the 2nd experimental group, probably due to inhibition of endogenous synthesis. Our experiments for the first time showed the results of activity of duodenal enzymes of chyme in the postprandial period after 60 and 120 min, which corresponds to complex reflex and humoral regulation of digestion in poultry.

## 5 Conclusion

The results of the research allow us to draw the following conclusions:

1. The enzyme preparation enhances intestinal digestion, improving digestion of feed protein for metabolism including egg formation, but high dose of the preparation (200 mg/kg of feed) disturbs the homeo-stasis of minerals critical for ~20–30% of daily calcium consumption per egg.
2. Enzyme preparation at a dose of 100 mg/kg of feed when added to the feed of laying hens provided the highest productivity, increasing the number of eggs laid during the period of the experiment by 25% more than in the control group, with a minimal decrease in egg weight (−1.4%). The 0.02% enzyme dose increased egg number by 12.5% compared with control but reduced mean egg mass by 2.5% (50.95 g → 49.7 g; *p* > 0.05). This suggests a dose-dependent effect: an optimal dose (experimental group 1) increases egg-laying frequency with a positive correlation between trypsin activity and egg production without compromising egg quality, while a high dose (experimental group 2) may cause metabolic overload, reducing egg weight (possibly due to a deficiency of shell minerals).

## Data Availability

The original contributions presented in the study are included in the article/supplementary material, further inquiries can be directed to the corresponding author.
